# High-Temperature Corrosion Behavior of Superheater Materials at 600 °C: Insights from Laboratory and Field Exposures

**DOI:** 10.1021/acs.energyfuels.4c04806

**Published:** 2024-12-12

**Authors:** Vicent Ssenteza, Maria Dolores Paz Olausson, Johan Eklund, Johanna Nockert, Jesper Liske, Torbjörn Jonsson

**Affiliations:** † Chalmers University of Technology, Kemivägen 10, 412 96 Gothenburg, Sweden; ‡ Valmet AB, Kruthusgatan 17, 411 04 Gothenburg, Sweden; § Kanthal AB, Sörkvarnsvägen 3, 734 40 Hallstahammar, Sweden

## Abstract

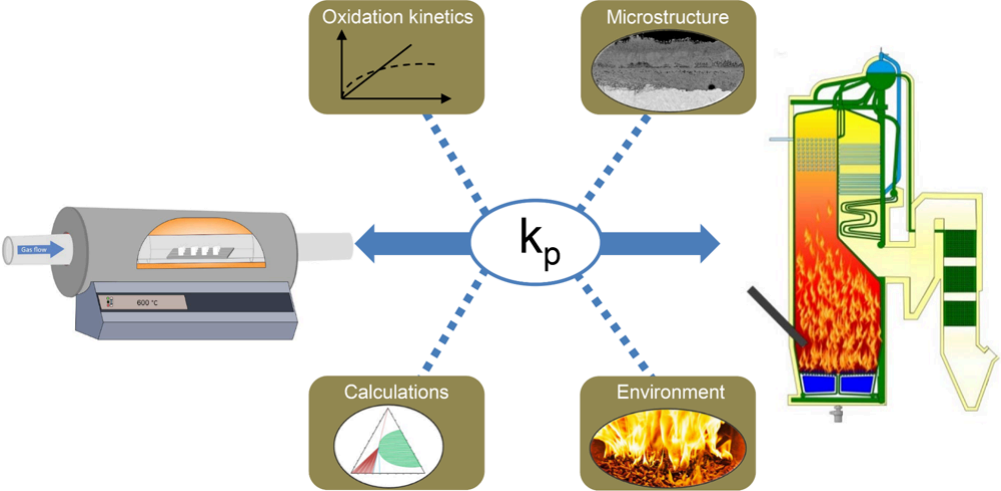

Combustion of biomass and waste results in release of corrosive species, such as alkali chlorides and water vapor, which accelerate the corrosion of superheaters in the boiler. To improve our understanding of alkali-induced corrosion, long-term corrosion investigations are needed. This study utilizes a systematic approach based on long-term corrosion studies (up to 8000 h) in a well-controlled laboratory environment to understand the corrosion behavior and protectiveness of oxide scales formed on a FeCr alloy (marginal chromia former) and three overlay weld coating systems (lean FeCrAl, FeCrAl, and Ni-based alloy) in a KCl-rich environment at 600 °C. The results show that all the alloys undergo fast breakaway corrosion and form duplex-layered scales consisting of outward- and inward-growing oxide scales. The marginal chromia former exhibits parabolic oxidation kinetics and forms Fe-rich oxides in the outer scale and mixed Fe- and Cr-oxides in the inner layer. These oxide microstructures are compared to the scales formed on probe-exposed samples in boilers, and similar microstructures are typically found after exposures. The FeCrAl coatings form Fe-rich oxides in the outer layer and Fe-, Cr-, and Al-oxides in the inner layer. All alloy systems except the Ni-based coating show corrosion rates in the boiler in good agreement with the laboratory test prediction. The Ni-based coating exhibits the slowest oxidation kinetics in the laboratory, forming thin oxide scales with Ni-rich oxides in the outer layer and Cr-rich oxides in the inner layers, while this is not the case in the waste-fired boiler.

## 1 Introduction

The use of renewable fuels, such as waste and biomass, for combined heat and power production has steadily been increasing in the last few decades. The main reason for this is the need for a reduction of both land filling and the net emission of CO_2_, for which the latter is considerably smaller when combusting biomass/waste compared to utilizing fossil fuels. However, the combustion of biomass and waste is well-known to result in a corrosive environment within the boiler due to the formation of, e.g., alkali salts and high levels of H_2_O(g), which accelerate the degradation of various components in the boiler, such as the superheater tubes (SH).^1−12^ Severe corrosion may lead to reduced material lifetime, particularly in the SH region, and costly unpredicted stops.^13−15^ To decrease the corrosion rate of the SH, the material temperature, and thereby also the steam temperature, has been decreased, resulting in a reduction of the electrical efficiency compared with boilers operated with fossil fuels. With operating conditions of most boiler systems restricted to <580 °C (final steam temperature), the electrical efficiency achieved is <40% compared to state-of-the-art coal fired boilers which is about 49%.^16^ An improvement of material/coating selection that enables operation at higher temperatures is crucial to increase the electrical efficiency. The key to this strategy is based on a good understanding of corrosion behavior.

Currently, the common choice of materials for SH tubes are low-alloyed steels^17^ due to their price and good mechanical properties. However, under harsh conditions, such as those found in the superheater region, these materials suffer accelerated corrosion, which is also the case for stainless steels. A promising material solution is the use of coatings for which a corrosion-resistant material is combined with a substrate with good mechanical properties. Over the last decades, several techniques have been developed to produce coatings that can be deployed on heat transfer surfaces to protect against corrosion, e.g., overlay weld, thermal spray, coextrusion, diffusion treatment, and laser cladding.^18^ Several studies have been conducted to understand the corrosion behavior of these coatings both in laboratory and boiler environments, as summarized by Kawahara^18^ and Wu et al.^19^ Despite this research, there are challenges with understanding and predicting material degradation in complex boiler environments. To increase the knowledge regarding superheater corrosion in biomass- and waste-fired boilers, a viable path is to perform long-term corrosion investigations in well-controlled laboratory environments for comparison with samples exposed in real boilers. However, such long-term laboratory exposures are scarce in these environments and in the relevant temperature range.

The aim of this investigation is to link laboratory observations of the high temperature corrosion behavior of different coating systems with field investigations. For this purpose, the study utilizes long-term corrosion exposures (up to 8000 h) performed in a well-controlled laboratory environment, simulating corrosive key components in biomass- and waste-fired boilers, to understand the corrosion behavior and protectiveness of oxide scales formed in harsh environments. The field exposures were performed in commercial biomass- and waste-fired boilers by utilizing corrosion probes and fixed installed materials. Postexposure analysis was performed by using advanced scanning electron microscopy coupled with energy dispersive X-ray spectroscopy (SEM/EDX) to characterize the corrosion products on sample cross sections prepared by using broad ion beam (BIB) milling.

## 2 Methodology

### 2.1 Material

For this investigation, four coatings from different material classes were used. A marginal chromia former (ferritic-martensitic stainless steel) supplied by Vallourec S.A, two FeCrAl coatings (a lean FeCrAl with 12.4 wt % Cr and a normal FeCrAl with 21 wt % Cr) supplied by Kanthal AB, and a Ni-based coating supplied by Alleima AB. The lean FeCrAl coating with low Cr (12.4 wt %) was selected to avoid embrittlement (i.e., the separation of Fe-rich α- and Cr-rich α′-phases) that occurs in ferritic stainless steels around 475 °C, while the higher Cr content may contribute to formation of more protective scale. The chemical compositions of the coatings are listed in Table 1. The same coatings were tested in both laboratory and boiler environments.

**Table 1:** Chemical Composition (wt %) of Materials Used in This Study

Coating	Cr	Ni	Al	Mo	Si	Mn	Fe
Marginal chromia former (lab)	10.5	0.4		0.6	0.6	0.8	Bal.
Marginal chromia former (field)	8–9.5	<0.04	–	0.85–1.05	0.2–0.5	0.3–0.6	Bal.
Lean FeCrAl	12.4	–	3.7		1.25	0.10	Bal.
FeCrAl	21	–	5	3	0.7	0.4	Bal.
Ni-based coating	21	Bal.	0.19	9.0	0.2	0.35	4.29

For the laboratory corrosion test, the coatings of dimensions 20 mm × 10 mm × 8 mm were produced by applying the material on one side of the substrate (the marginal chromia former) through Mech-MIG, with Pulse Multi Control. The welding parameters were 179A current and 25 V voltage. The rest of the sides of the samples were painted with an Al slurry coating to prevent the sides from corrosion during exposure.

For the field corrosion test, ring samples were produced with a diameter of 38 mm and width of 11 mm. The marginal chromia former tube was overlay welded with the different coatings before cutting the tube into sample rings. The welding parameters were the same as those for the laboratory samples. The surface of the sample rings was machined after welding to remove the shape of the welding cords to make it possible to calculate the material loss after the exposure.

### 2.2 Sample Preparation and Exposures

Prior to the laboratory corrosion tests, 2 mg/cm^2^ KCl(s) was predeposited on the sample surfaces by spraying a KCl solution (80 vol % ethanol +20 vol % water) on the samples under a continuous flow of warm air to speed up the drying of the salt. The weight of the samples was taken occasionally to monitor the amount of salt deposited. Thereafter, the samples were kept in a desiccator for 24 h for the salt to dry completely and a final weight was taken just before exposure. The samples were then placed on alumina sample holders and exposed in a horizontal tube furnace with an environment consisting of 5% O_2_ + 20% H_2_O + N_2_ (Bal.) at 600 °C for 168, 500, 1000, and 8000 h. The gas flow through the system was set at 0.1 cm/s.

The field exposures were carried out in three different boilers. All of the exposures were performed in the superheater region using an air-cooled probe consisting of two temperature zones, controlled using thermocouples (one for each temperature zone), and the exposure temperature was set to 600 °C in both zones. The marginal chromia former and the FeCrAl were exposed for 3000 h in a bubbling fluidized bed (BFB) boiler. The combusted fuel was a solid fuel mixture that included paper factory waste, forestry waste, industrial wood waste, and municipal wood residues. Natural gas is used as the starting fuel and as an additional support fuel. The lean FeCrAl alloy was exposed for 2000 h in another BFB boiler with wood chip-combusted fuel. Finally, the Ni-based alloy was exposed in a waste-fired grate boiler for 1000 h.

### 2.3 Postexposure Analysis

Imaging and chemical analysis of the corrosion products was performed using a FEI Quanta 200 scanning electron microscopy (SEM) equipped with a field emission electron gun (FEG) and an Oxford EDX system. The SEM/EDX system was operated using accelerating voltages of 10 and 20 kV. Prior to analysis, cross sections of laboratory samples were prepared using broad ion beam milling with a Gatan PECS II system operated at 8 kV. The oxide thicknesses were measured using the ImageJ software on SEM-BSE cross-sectional images. The field samples were cast in epoxy, cut, and polished prior to the SEM/EDX investigation. The samples were cast in epoxy resin by putting them into a mold, which were subjected to a 10-bar pressure to avoid the formation of bubbles during the hardening of the resin. The hardening time was fixed at 24 h. After the hardening of the epoxy resin was complete, the samples were cut by using a silicon carbide disc and a lubricant without any water due to the delicate corrosion products. The samples were then polished dry with SiC paper (up to P4000). The cross section was coated with gold to avoid charging in the SEM. The oxide thickness was measured with SEM at 8 different locations by turning the sample ring 45° each time.

The concentrations of Cl^–^ and SO_4_^2–^ were determined using ion chromatography (IC) (Dionex ICS-90 system with an IonPac AS4A-SC analytic column). A solution of 1.8 mM NaHCO_3_/1.7 mM NaHCO_3_ was used as eluent with a flow rate of 2 mL/min, and the results presented as mass percentage (amount of ions/100 g deposit).

## 3 Results

In this section, the results of corrosion tests in the laboratory environment are presented first, followed by the results from the boiler.

### 3.1 Corrosion Tests in Laboratory Environment

#### Oxidation Kinetics in Laboratory

**Figure 1:**
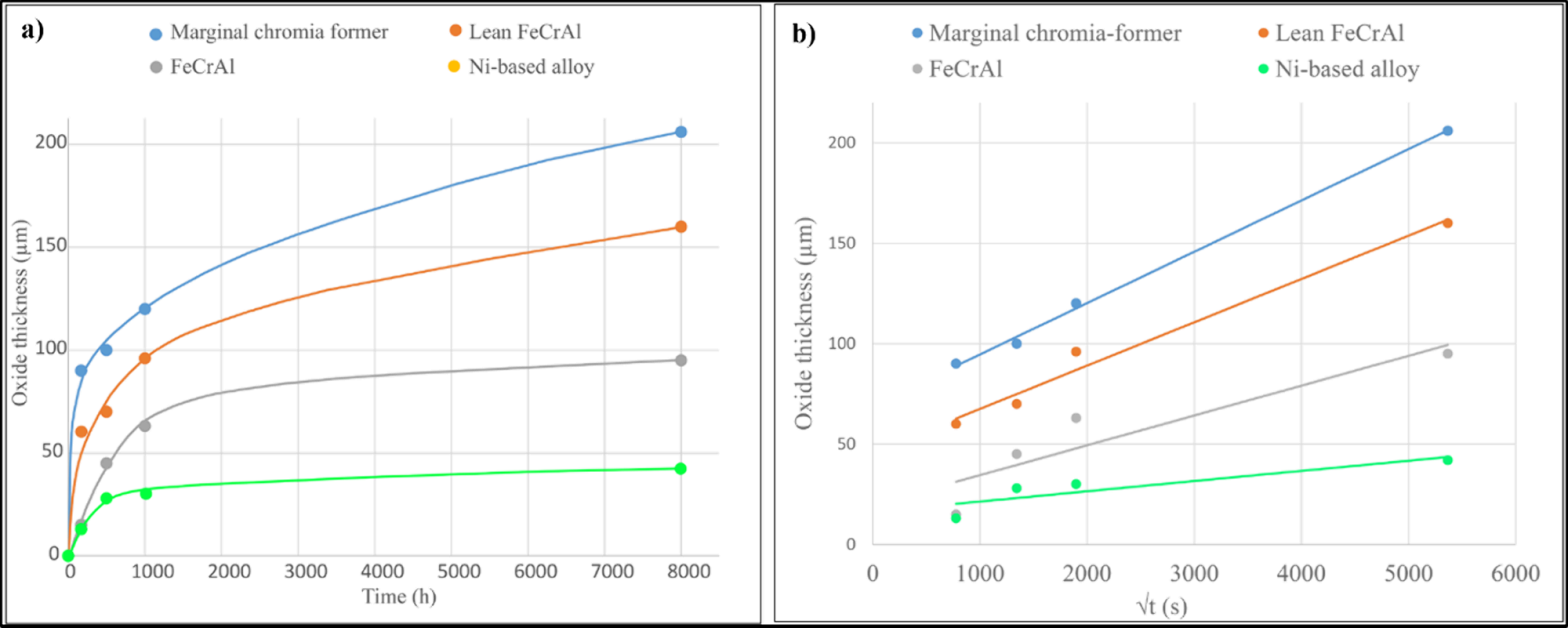
Oxidation kinetics based on oxide thickness measurements after exposure in laboratory environment (O_2_+H_2_O+KCl) at 600 °C. The oxide thickness for the samples tested in laboratory was measured from the SEM cross-section images using ImageJ software.

The oxide growth kinetics were evaluated by means of the oxide thickness. Figure 1a shows the oxidation kinetics based on the measured average oxide thickness for all the tested coatings in the laboratory environment (O_2_+H_2_O+KCl) at 600 °C. The thickness measurements were performed on wide SEM-BSE cross-section images prepared using ion milling. Generally, all the coatings exhibit varying oxide growth kinetics. In order to be able to assess corrosion performance in the investigated environments, the parabolic rate constants (*k*_p_) were determined from the long-term laboratory exposures using eq 1, where the measured average oxide thicknesses (μm) are plotted against the square root of exposure time (in seconds), see Figure 1b. The obtained *k*_p_ values enable recalculation of the predicted oxide thickness for the field samples at any given exposure time. For this study, well-controlled laboratory conditions provide oxidation kinetics with reliable data that can be correlated to field exposures.
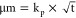
1

The marginal chromia former exhibits the fastest oxide growth, from ∼90 to ∼206 μm after 168 and 8000 h, respectively. According to Figure 1b, which shows the thickness plotted against square of time, the marginal chromia former exhibits a parabolic oxidation behavior with very small deviation for all exposure times. The slope of the plotted line in Figure 1b gives the *k*_p_ value and is calculated to be 0.0256 μm^2^/s. The lean Feral alloy follows a similar trend, while the deviation from strictly parabolic kinetics is larger, with a calculated *k*_p_ value of 0.0216 μm^2^/s. This material exhibits slightly higher initial oxide growth, which gradually slows down at a faster rate than the marginal chromia former.

The FeCrAl alloy and especially the Ni-based coating display the slowest oxidation kinetics, with calculated *k*_p_ values of 0.0149 and 0.0051 μm^2^/s, respectively. The oxide thickness ranges from 13 to 42 μm (Ni based alloy) after the longest exposure time (8000 h). The thickness/sqrt(t) plots in Figure 1b indicate a very large deviation from strictly parabolic behavior and very strong subparabolic kinetics. This could be interpreted as two different corrosion regimes of oxidation kinetics being at play. Initially, following breakaway oxidation, a faster oxidation kinetics is observed, while the kinetics slows down with exposure time.

#### Oxide Microstructure

**Figure 2:**
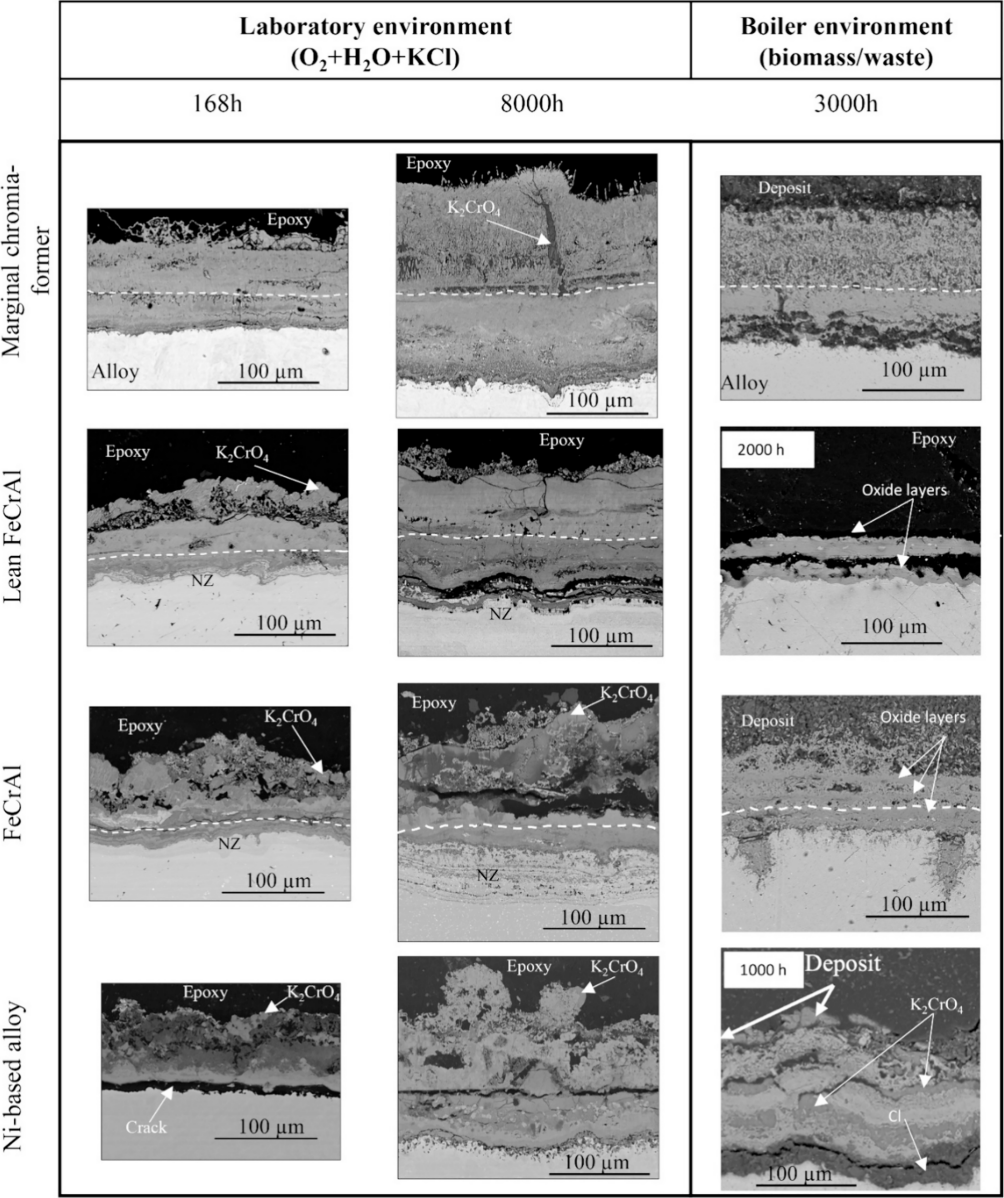
SEM-BSE cross-section images showing the oxide microstructure after exposure in laboratory environment (O_2_+H_2_O+KCl) and biomass- and waste-fired boiler environments at 600 °C.

Figure 2 shows SEM-BSE cross-sectional images of the coatings investigated, displaying the oxide microstructure after exposure in the laboratory and boiler environments. All the alloys have undergone breakaway corrosion after the shortest exposure time (168 h), forming multilayered oxide scales.

The marginal chromia former forms thick and dense oxide scales that are well-adherent to the metal surface with a microstructure that can be interpreted as outward- and inward-growing oxide scales. The interface between the scales is marked by the white dashed lines and is considered to be the original surface before the oxidation. The interpretation is based on SEM/EDX elemental analysis, which shows that the outward-growing scales are Fe-rich oxides, while the inward-growing scales exhibit slight Cr enrichment, as shown in Table 2. The outward-growing scale results from the outward diffusion of Fe ions, while the inward-growing scale results from the inward-diffusion of anions (O^2–^). Large K_2_CrO_4_ particles are in addition embedded within the outward-growing oxide after the 8000 h exposures. Trace amounts of K and Cl (<1 at %) could be detected in the outward-growing and inward-growing scales, respectively. Indications of a very thin more Cr-rich layer was observed after 8000 h (marked in Figure 2).

**Table 2:** Average Elemental Compositions of the Oxide Scales (at % cation)

	Laboratory exposure (168 h)							
	Outward-growing scale				Inward-growing scale			
Coating	Fe	Cr	Al	Ni	Fe	Cr	Al	Ni
Marginal chromia former	100				75	25		
Lean FeCrAl	80	15	5		40	40	20	
FeCrAl	70	20	10		25	55	20	
Ni-based coating	15	5		80		80		20

	Laboratory exposure (8000 h)							
	Outward-growing scale				Inward-growing scale			
Coating	Fe	Cr	Al	Ni	Fe	Cr	Al	Ni
Marginal chromia former	100				65	35		
Lean FeCrAl	100				30	55	15	
FeCrAl	100				20	70	10	
Ni-based coating	20	10		70	5	90		5

	Field exposure (3000/2000/1000 h)							
	Outward-growing scale				Inward-growing scale			
Coating	Fe	Cr	Al	Ni	Fe	Cr	Al	Ni
Marginal chromia former	100				80	20		
Lean FeCrAl					80	15	5	
FeCrAl	90		10		20	50	30	
Ni-based coating	5	10		85	Not detected			

The FeCrAl coatings (lean FeCrAl and FeCrAl) exhibit similar scale microstructural features in both environments, where they form dense double-layered scales, i.e., outward- and inward-growing scales (the interface is marked with white dashed lines in Figure 2). Initially (168 h), the outward-growing scales are composed of Fe-rich oxides, while the inner scales are composed of mixed Fe-, Cr-, and Al-oxides according to SEM/EDX results (see Table 2). After 8000 h of exposure, the outward-growing scales are still composed of Fe-rich oxide, while the inner scales have become enriched in Cr (55 at % and 70 at % for lean FeCrAl and FeCrAl respectively). A large crack is observed at the bottom of the oxide scale on the lean FeCrAl coating after 8000 h-exposure. After all exposures, nitridation zones (NZ) have formed beneath the scales of both coatings, as indicated by the presence of N and Al in these regions. At the surface, K_2_CrO_4_ particles are detected on both coatings. Trace amounts (<1 at %) of K and Cl are detected in the outer and inner scales, respectively.

The Ni-based coating formed a thin and dense oxide scale. The scale is double-layered, consisting of outward- and inward-growing layers. The scale formed after 168 h of exposure had delaminated, possibly during postexposure sample preparation. However, the scale on the sample exposed for 8000 h was adherent to the coating with void formation beneath the scale. According to SEM/EDX analysis, the outer oxides are composed of mainly Ni, while the inner scales are enriched in Cr (80 at. % Cr and 90 at. % Cr after 168 and 8000 h of exposure respectively). K_2_CrO_4_ particles could again be observed at the surface. A summary of the key chemical composition of the oxide scales formed during laboratory exposures is presented in Table 2.

### 3.2 Corrosion Tests in Boiler Environment

#### Deposit Chemistry

The deposits over the marginal chromia former and the FeCrAl samples were collected after 3000 h of exposure in the biomass-fired boiler and analyzed using IC. The results showed that the sample deposit contained less than 0.01 wt % Cl^–^ and 8 wt % SO_4_^2–^. The deposit from the lean FeCrAl contained 0.02–0.1 wt % Cl^–^ and 12–20 wt % SO_4_^2–^ after exposure for 2000 h. The deposit from the Ni-based sample was collected after 1000 h exposure in the waste fired boiler and contained 6.5 wt % Cl^–^ and 4 wt % SO_4_^2–^.

#### Oxide Thickness after Field Exposures

Table 3 shows the measured and calculated oxide thicknesses for the coatings after exposure in commercial boilers. The calculated thickness is based on the *k*_p_ values obtained from oxidation kinetics in laboratory environment (see Figure 1). The marginal chromia former exhibits an oxide thickness of 150 μm after 3000 h of exposure in boiler, which is in good agreement with the calculated thickness (148 μm). The calculated thickness for lean FeCrAl is 119 μm. It may be noted that only the inward-growing layer (50 μm) of the oxide scale was recovered after exposure and not the outward-growing layer. Based on the normal distribution of inward/outward growing scales, the predicted total oxide scale would be 100 μm. In the case of the FeCrAl, the calculated and measured thickness were 69 and 70 μm, respectively, after exposure for 3000 h. For the Ni-based alloy, the calculated thickness was 25 μm, while the measured thickness was 120 μm after 1000 h of exposure.

**Table 3:** Measured and Calculated Oxide Thicknesses for the Investigated Coatings

Coating	Laboratory environment, Calculated oxide thickness (μm)	Boiler environment, Average measured oxide thickness (μm)	Exposure time (h)
Marginal chromia former	148	150	3000
Lean FeCrAl	119	50 (inner)/100 (predicted)	2000
FeCrAl	69	70	3000
Ni-based coating	25	120	1000

#### Oxide Microstructure

The ring samples exposed in the superheater region of the different boilers form oxide scales of varying thickness depending on the direction of incoming flue-gas, i.e., wind side or lee side. The wind side of the sample may be exposed to a combination of erosion and corrosion, while the lee side is generally only exposed to corrosion. Since the purpose of this study is to understand the corrosion behavior of the different coatings in the boiler environment, the oxide thickness of interest corresponds to the lee side of the rings to best correlate to the simulated laboratory environment, i.e., without erosion. All of the alloys have undergone breakaway corrosion after the corresponding exposure times.

The marginal chromia former exposed for 3000 h in the biomass-fired boiler formed thick and dense oxide scales with a similar microstructure as the samples exposed in the laboratory, consisting of outward- and inward-growing oxide scales (with the interface between these scales marked by the white dashed line), see Figure 2. The adhesion of the inward-growing scale is very poor. The contraction/expansion forces from the epoxy casting are enough to separate the oxide from the substrate during the hardening of the epoxy. According to SEM/EDX analysis, the outward-growing scale is composed of Fe-rich oxides, while the inward-growing scale is rich in Fe and Cr (Table 2). The measured thickness is 150 μm which is in good agreement with the calculated thickness based on the *k*_p_ value from the laboratory exposures, see Table 3. The surface of the sample is covered by a thick deposit, which according to SEM/EDX analysis is mainly composed of CaSO_4_ and K_2_SO_4_. Very small concentrations of chlorine were detected on the top of the oxide scale surface, not in contact with the metal substrate. These results are consistent with the IC measurements where a negligible amount of Cl was found.

The lean FeCrAl alloy presents a measured average inward-growing oxide scale thickness of 50 μm after 2000 h of exposure and a predicted total thickness of 100 μm. The scale contains horizontal cracks and is mainly composed of mixed Fe-, Cr-, and Al-oxides (Table 2), which is typical of the inward-growing oxide scales observed in FeCrAl alloys after exposure in biomass/waste environments. According to SEM/EDX analysis, no outward growing oxide could be detected. It is possible that the outer scale spalled off during postexposure treatment and that only the inward-growing oxide scale was retained. Due to this effect, the 50 μm thick oxide scale does not represent the whole thickness of the oxide. No Cl signal has been detected.

The FeCrAl coating exhibits microstructural features similar to those for the laboratory samples, where it forms a dense double-layered scale, i.e., outward- and inward-growing scales that are well attached to the metal. The oxide scale is roughly 70 μm thick. The outward-growing scale is mixed with the deposit and has a layered structure. The outer scale is Fe rich (90 at. % Fe and 10 at. % Cr) while the inner scale is Cr rich (20 at. % Fe, 50 at. % Cr, and 30 at % Al). Furthermore, the coating experienced grain boundary attack to a depth of 100 μm into the coating. The deposit composition is the same as for the chromia former, and no chlorine signal has been detected either with EDX or IC.

The Ni-based coating formed a thin and dense oxide scale. After 1000 h of exposure in the waste-fired boiler environment, the coating has formed a 120 μm thick, dual-layered oxide scale. The surface is covered with a deposit. SEM/EDX analysis revealed the presence of large K_2_CrO_4_ particles embedded in oxide layers. Large amounts of Cl up to 30% were detected within the oxide.

## 4 Discussion

It is well-known that the combustion of biomass and waste results in the formation of corrosion species, e.g., alkali salts, which lead to faster degradation of superheater steels. Several alloy systems representing potential material solutions to the corrosion challenge have been investigated. However, all alloy systems are expected to undergo fast breakaway oxidation in this very corrosive environment.^1,8^ Thus, the corrosion performance is expected to mainly depend on the secondary protection; i.e., the scale formed after breakaway oxidation.^3^ To better understand the corrosion behavior of these materials/coatings in the biomass/waste-fired boiler environments, this study utilizes long-term corrosion exposures in a well-controlled laboratory setup, which introduces fast breakaway corrosion. It is worth noting that the corrosion assessment approach taken in this study considers the propagation phase and not the initiation phase. Several short-term studies (≤168 h) have investigated the initiation of alkali chlorine-induced corrosion in detail, focusing on oxidation kinetics,^20,21^ breakdown of primary oxide,^1,22,23^ and influence of environment on initial oxide formation.^24,25^ The breakaway oxidation is generally characterized by fast oxidation kinetics with local differences.^26^ The current study setup considers the long-term post-breakaway phase, and the calculations, i.e., the calculated *k*_p_ values, are focused on the long exposure times, see Figure 1. The initial phase may have a different *k*_p_ value originating from an oxide formed before 168 h.

### 4.1 Corrosion Performance

Generally, all the coatings as well as the marginal chromia former undergo breakaway oxidation after the shortest exposure time of this study (168 h) in the laboratory environment and form multilayered oxide scales that consist of outward and inward-growing oxide. This is in line with earlier investigations of these types of material systems in this environment, see, e.g., refs (3 and 27). According to the oxidation kinetics of laboratory exposed samples, the coatings/marginal chromia former display varying rates of oxide growth with the marginal chromia former exhibiting the fastest oxide growth rate, and the Ni-based alloy exhibiting the slowest growth rate since no difference in incubation time to breakaway could be observed. This supports previous results, which show that the difference in oxide growth kinetics is related to the performance of the secondary corrosion regime and can be correlated to the alloy composition where the high-alloyed steels, i.e., FeCrAl and Ni-based alloys, display slower growth rates.

The low Cr-containing alloys, i.e., the marginal chromia former and the lean FeCrAl alloy, display fast oxidation kinetics, resulting in thick oxide scales. According to elemental distribution from the SEM/EDX analysis, the alloys form Fe-rich outward-growing scales (or Ni-rich outer scale in the case of Ni-based alloy) and Cr/Al containing inward-growing scales. The scale microstructures and elemental distributions, together with the growth kinetics, indicate diffusion-controlled mechanism where the microstructure of the oxide scales is determined by the diffusivity of ions through the scales.^28^ The results of this study show that despite the presence of large amounts of Cl detected in some samples there is no indication of the active oxidation mechanism, driven by gaseous transport of Cl_2_(g) and MeCl_*x*_(g), whereas the transport of O_2_(g) is prohibited, which has been suggested in literature.^29^ The marginal chromia former displays parabolic kinetics with a very small deviation. The lean FeCrAl coating follows a pattern similar to that of the marginal chromia former. However, it slows down faster, which indicates the formation of a Cr/Al-rich protective scale closer to the metal/scale interface, i.e., “healing layer”, after shorter exposure time than the marginal chromia former (see Figure 2). Microstructural investigation of the inward-growing scale formed on the lean FeCrAl coating exposed in the boiler in addition reveals a thinner scale with the predicted inward growing scale; i.e., the outward growing scale has probably spalled off during handling.

The two coating systems that display very subparabolic behavior in the laboratory exposure, e.g., the FeCrAl and the Ni-based alloys, form thin scales because of the presence of a distinct healing layer after shorter time in the laboratory exposure. Such an early transition to slow kinetics could be explained by the high Cr content in the coatings that lead to improved corrosion resistance as reported in earlier study, see, e.g., ref (30).

Comparably, the calculated oxide thickness for 1000–3000 h under laboratory conditions are in good agreement, except for the Ni-based alloy, with the average measured oxide thickness on samples exposed in the biomass/waste-fired boiler environments (see Table 3). It may be noted that the corrosion rate for the Ni-based alloy in the boiler is faster than in the laboratory for the systems relying on the improved protection in the form of a healing layer. This is in good agreement with the microstructural investigation where the healing layer is absent in the field exposed sample. This may be caused by the large amounts of Cl found in the metal/oxide interface. Several studies have previously reported high corrosion rates for samples exposed in boiler environments, see, e.g., refs (31−33).The main contributing factors were suggested to be flue gas temperature, flue gas velocity, position of the samples, unplanned stops which lead to unexpected thermal cycling, and thickness of the deposit, which can contribute to higher rates of corrosion in the boiler. While the samples in the laboratory environment are exposed to a continuous and controlled environment, the field samples can suffer changes in the flue gas velocity or flue gas temperature depending on the load of the boiler. Those changes lead to higher temperature gradients in the samples, accelerating the kinetics of oxide formation. It is common that the outer part of the oxide scale falls off during exposure, due to thick deposits or poor adhesion of the oxide (see the marginal chromia former after 3000 h of exposure in the boiler environment in Figure 2). The surface of the metal remains unprotected, and the oxide scale formation process starts again. Nevertheless, even though the oxide scales are thicker on one of the field exposed samples, the corrosion mechanism is argued to be the same in the two environments, allowing a fair comparison between the oxide scales and simplifying the characterization of the samples.

### 4.2 Oxide Microstructure

The microstructural investigation of the oxide scales reveals that all the alloys have undergone breakaway corrosion and formed multilayered oxide scales that can be interpreted as the outward- and inward-growing oxide scales (see Figure 2). The outward-growing scales are Fe-rich oxides or Ni-rich oxides in the case of the Ni-based alloy. SEM/EDX analysis of the corrosion products revealed the formation of K_2_CrO_4_ particles at the surface of all the investigated materials. Such corrosion products are associated with the breakaway phenomenon, where the alkali salts react with the protective scales (Cr_2_O_3_) leading to fast-growing and less protective Fe-rich oxide scales.^34^ The inner scales are more complex and composed of different elements depending on the alloy.

The marginal chromia former, with the highest rate of oxidation, forms very thick oxides, most probably due to the small Cr enrichment that is observed in the inner scale (25 at. % after 168 h and 35 at. % after 8000 h respectively). This is supported by the oxide microstructure where only indications of a healing layer with somewhat higher Cr content could be observed after 8000 h in the laboratory. Instead, this type of alloy is known to form a zone of internal oxidation at the corrosion front offering very limited diffusion protection of iron.^3^ Thermodynamic calculations, which indicate that this alloy should form mostly the spinel-type/internal oxidation and a minor fraction of corundum (∼20%) in the inward-growing scale,^30^ further support this concept. The microstructure is very similar after exposure in the boiler, and the calculated thickness (based on the *k*_p_ value from the laboratory exposures) predicts the thickness with high accuracy (see Figure 2 and Table 3) indicating that the same oxidation mechanisms are at play despite the very complex environment in the commercial boiler. When this alloy is exposed to alkali chlorides, it is unable to form and sustain a protective Cr-rich inner scale; instead it exhibits similar microstructural features as low-alloyed steels, i.e., formation of thick multilayered Fe-rich oxides.^35^

Furthermore, the role of alloying elements on corrosion behavior of alloys is noted on the rest of the investigated coatings, i.e., lean FeCrA, FeCrAl, and Ni-based alloys. The FeCrAl alloys contain Al, which has been shown to have a positive effect on the corrosion behavior after breakaway corrosion by preventing internal oxidation and enabling formation of more protective inner scales.^27^ Although these alloys do not form protective α-alumina scales at intermediate high temperatures (600 °C), the resulting mixed Fe-, Cr-, and Al-oxides in the inner scale offers better corrosion resistance than the Fe- and Cr-oxide formed by the marginal chromia former. The results of this study clearly show that the FeCrAl coating exhibits better corrosion resistance than lean FeCrAl in both investigated environments. This difference in corrosion performance is attributed to the high Cr content of the FeCrAl alloy, which leads to faster formation of a healing layer as indicated by subparabolic kinetics, see Figure 1. In addition, the SEM/EDX analysis clearly showed that the FeCrAl alloy experiences Cr enrichment in the inner scales reaching up to 70 at % after 8000 h compared to 55 at % for the lean FeCrAl. Such a high Cr activity in the inward-growing scale of the FeCrAl alloy indicates the formation of a more protective corundum-type oxide (healing layer), which is predicted by the thermodynamic calculations.^30^ When exposed in the boiler, both FeCrAl alloys formed thick oxide scales. SEM/EDX analysis revealed that the inner scale of the FeCrAl coating is richer in Cr (50 atom %) than for the lean FeCrAl (15 at % Cr). Observably, the FeCrAl coating experiences repetitive breakaway corrosion leading to formation of an alternating Cr-rich scale (healing oxide layer)/Fe-rich scale, possibly due to fluctuating conditions in the boiler. This process repeats over the whole exposure, generating a thicker layered oxide scale. On the contrary, this effect is not observed in laboratory exposures where only one Cr-rich oxide layer appears close to the scale/coating interface due to the very stable conditions in the laboratory furnace environment.

The Ni-based coating exhibits the slowest oxidation kinetics (subparabolic) and has therefore formed the thinnest oxide scales during the laboratory exposures. The subparabolic kinetics indicates that this coating forms a protective inner scale (healing layer) at an early stage of the corrosion process as revealed by the Cr-rich inner scale (80 at. % Cr) after only 168 h. The alloy thereby quickly transitions into a slower oxidation stage, where the protective scale is protected from the alkali-rich environment by the Fe-rich scale formed directly after breakaway oxidation. This mechanism can be explained by the slow inward diffusion of oxygen in combination with a high Cr activity in the bulk, which promotes the faster formation of the predicted corundum-type oxide. The fast outward diffusion of Cr is evident through the accumulated pores at the coating/scale interface because of the Kirkendall effect.^36^ In contrast, the Ni-based alloy forms a thicker oxide scale in the waste-fired boiler environment, indicating a faster growth rate. In this case, both the Cl content (6.5% Cl in the deposit) and the flue gas temperature (close to 900 °C in this boiler) play crucial roles in accelerating the corrosion rate. The Cl penetrates the scale and reaches the alloy surface, forming metal chlorides, which debilitates the oxide scale and accelerates the corrosion rate by disturbing the formation of a healing layer, see Figure. 2.

It is well-known that Cl accelerates the corrosion of alloys at intermediate temperatures (600 °C). It has been suggested that gaseous Cl_2_ is transported to the metal–scale interface, where it forms volatile metal chloride species. The formed metal chlorides are then suggested to be transported as gas molecules through a porous oxide scale toward the surface where they are converted to metal oxides upon reaching regions of high oxygen partial pressure.^37−39^ In this study, SEM/EDX analysis of the Ni-based alloy shows Cl-rich regions close to the metal–scale interface, which could originate from the formed metal chlorides. However, the microstructure as well as the oxidation kinetics strongly suggest a diffusion-controlled mechanism.^40^ Further microstructural analysis revealed the formation of K_2_CrO_4_ particles between the oxide layers. Considering the thickness and dense microstructure of the oxide layers in combination with the K_2_CrO_4_ layers, it is unlikely that the active oxidation mechanisms is the main degradation mechanism in this case, but rather a combination of K_2_CrO_4_ formation and diffusion-controlled growth mechanism. It has been shown that the reaction between K- and the Cr-rich oxide scale leads to fast degradation, depleting the scale of Cr.^34^ The diffusion of chlorine through the scale is instead suggested to occur via ion diffusion of Cl^–^, as postulated in the electrochemical mechanism.^41^ The results of microstructural investigations in this study indicate that the formation of a healing layer is the key to improving corrosion resistance in harsh environments, which is in line with previous studies that have investigated oxide scales formed after breakaway oxidation.^30^

## 5 Conclusion

Four potential material/coating systems have been investigated through long-term exposures under a well-controlled laboratory environment and in commercial boilers. All the alloys experience breakaway corrosion and form multilayered scales that consist of outward- and inward-growing oxide, i.e., diffusion-controlled kinetics. Corrosion investigation under laboratory conditions provides reliable kinetic data that can be correlated to field investigations.

The corrosion rate is in one case faster in the biomass/waste boiler environment than under laboratory conditions. The results indicate a diffusion-controlled growth mechanism and breakaway oxidation caused by K_2_CrO_4_ formation. The corrosion protection is governed by the formation of a healing layer at the bottom of the scale formed after breakaway oxidation and depends on the alloy composition. The marginal chromia former exhibits parabolic oxidation kinetics and forms Fe-rich oxides in the outer layer and mixed Fe- and Cr-oxide in the inner layer. The FeCrAl alloys displayed intermediate oxidation rates in the laboratory environment and formed Fe-rich oxides in outer scales and Fe-, Cr-, and Al-oxides in the inner scales. The Ni-based alloy displayed subparabolic oxidation behavior and formed thin oxide scales that are Ni rich in the outer layer and Cr rich in the inner layer during the laboratory exposure.

## Data Availability

Data will be made available on request.
